# Emotions, Alexithymia, and Emotion Regulation in Patients With Psoriasis

**DOI:** 10.3389/fpsyg.2020.00836

**Published:** 2020-05-19

**Authors:** Maria Serena Panasiti, Giorgia Ponsi, Cristiano Violani

**Affiliations:** ^1^Department of Psychology, “Sapienza University of Rome,” Rome, Italy; ^2^Social Neuroscience Laboratory, IRCCS Fondazione Santa Lucia, Rome, Italy

**Keywords:** psoriasis, emotional reactivity, alexithymia, emotion regulation, stress

## Abstract

Psoriasis is a chronic dermatological condition that is frequently associated with problematic patterns of emotional reactivity (the way in which patients react to stimuli), alexithymia (their ability to recognize and label the emotional reaction), and emotion regulation (the ability to enhance or reduce their own emotional reaction). A research in the peer-reviewed scientific literature was conducted in order to identify articles describing the association of psoriasis and affective problems. In particular, we first evaluate studies that have investigated abnormal emotional reactivity (in terms of duration, frequency, or type of the experienced emotions) and its impact on patients’ quality of life; next, we review the role of alexithymia and emotion regulation in modulating the relationship between emotional reactivity and quality of life in this population. From a critical analysis of the reviewed studies, we highlight that altered emotional processing might be particularly important in the characterization of this condition. In particular, we show that this condition is related to an emotional reactivity characterized by negative emotions that have a stronger impact on patients’ quality of life when emotion regulation abilities are weak, especially if patients have alexithymia. Finally, we present suggestions for future directions in both clinical and research fields.

## Introduction

Psoriasis is a chronic inflammatory skin disease affecting approximately 2% of the population (Schmid-Ott et al., 2007) and characterized by cutaneous lesions that may appear on any part of the body. This condition can be very challenging and has such a strong impact on patients’ physical appearance in that embarrassment over appearance is rated as the most debilitating feature of the disease (Vardy et al., 2002). Psychological stress, in turn, has a negative impact on psoriasis symptoms leading to a self-perpetuating mechanism that might be difficult to interrupt (Basavaraj et al., 2011). In such a scenario, emotional reactivity (i.e., the emotional response provoked by the perception and the valuation of a given situation; Gross and Jazaieri, 2014) and emotion regulation (i.e., the ability to modify the perceived emotion in terms of its quality, intensity, or duration; Gross and Jazaieri, 2014) become particularly crucial. Importantly, the way in which we experience and regulate our emotions is strictly dependent on the ability to recognize and distinguish them from other bodily sensations (Chen et al., 2011), thus, deficit in such domain (i.e., alexithymia) can also worsen the affective experience of psoriasis patients.

In what follows, we provide a review of the literature tapping into emotional processing in psoriasis with the aim of characterizing it in terms of emotional reactivity, alexithymia, and emotion regulation. Even though these constructs can be correlated with each other, here, we highlight how their abnormal functioning is associated with different dermatological, psychological, or life quality outcomes. Finally, we discuss the implications for clinical practice and research.

## Methods

We conducted a search of PubMed’s database of articles containing the word “psoriasis” and one of the following terms: emotional reactivity, alexithymia, social exclusion, stigmatization, stress, anxiety, depression, and emotion regulation. Additional records were identified through manual searches of references of identified articles. Thirty-seven studies were selected (see Table 1).

**TABLE 1:** Description of the studies identified by the review.

**N**	**Authors Year**	**Type of study**	**Number of patients**	**Number of control participants**	**Emotional reactivity/regulation/alexithymia/stress**	**Measures behavioral/self-report**	**Outcome**
1	Cepuch et al., 2014	Cross-sectional	105	Absent	Emo	Self-report	High prevalence of anxiety
2	Pujol et al., 2013	Longitudinal (2 sessions)	164	Absent	Emo	Self-report	Anxiety is higher in women and correlates with the severity of the disease
3	Innamorati et al., 2018	Cross-sectional	50	50	Emo	Self-report	Higher anxiety and depression than controls
4	Vardy et al., 2002	Cross-sectional	100	100	Emo	Self-report	In psoriasis, the relationship between disease severity and quality of life is mediated by the feeling of stigmatization
5	Łakuta et al., 2017	Cross-sectional	148	Absent	Emo	Self-report	Stigmatization seems to be the most powerful predictor of depressive symptoms in these patients
6	Richards et al., 2001	Cross-sectional	115	Absent	Emo	Self-report	Stigmatization is related to psychological distress and degree of disability
7	Ginsburg and Link, 1989	Cross-sectional	100	Absent	Emo	Self-report	Stigmatization is higher when psoriasis has an early onset and the extent of bleeding is wider and feeling of rejection is stronger
8	Ginsburg and Link, 1993	Cross-sectional	100	Absent	Emo	Self-report	Stigmatization interferes with work and daily activities
9	van Beugen et al., 2017	Cross-sectional	514	Absent	Emo	Self-report	Stigmatization was associated with higher impact on daily life; lower education; higher disease visibility, severity, and duration; higher levels of social inhibition; having a type D personality; and not having a partner
10	Hrehorów et al., 2012	Cross-sectional	102	Absent	Emo	Self-report	Stigmatization is assiciated with: pruritus intensity, stress, depressive symptoms and lower quality of life
11	Ponsi et al., 2019	Cross-sectional	16	17	Emo ER	Behavioral and physiological	Higher sympathetic activity during social exclusion which brings to higher need for social reconnection
12	Lahousen et al., 2016	Cross-sectional	171	171	Emo	Self-report	Shame and disgust correlated with a less positive evaluation of being touched by their parents when they were kids
13	van Beugen et al., 2016	Cross-sectional	50 + 50 (significant ones psoriasis)	50 (alopecia) 50 (significant ones alopecia) 50 controls	Emo	Behavioral	Patients with psoriasis and their significant ones avoid disgusted faces more than controls
14	Sampogna et al., 2012	Cross-sectional	936	Absent	Emo	Self-report	Shame is higher in women than men. Shame and anger are more frequent in patients with low level of education
15	Jensen et al., 2016	Cross-sectional	42511	Reference population: 4724748	Emo	Clinical diagnosis	Developing depression after psoriasis is mediated by the presence of other comorbidities
16	Wojtyna et al., 2017	Cross-sectional	219	Absent	Emo	Self-report	Depression is predicted by: psychological distress, negative beliefs about one’s own appearance, and lower levels of emotional and social support
17	Pompili et al., 2016	Cross-sectional	112	77 (melanoma) 53 (allergy)	Emo	Self-report	Psoriasis is more frequently associated with suicidal ideation and attempt
18	Aydin et al., 2017	Cross-sectional	85	86 (healthy)	Emo	Self-report	Higher anger related to lower self-esteem
19	Matussek et al., 1985	Cross-sectional	38	113 (depression) 32 (healthy)	Emo	Self-report	High in outward aggression and low in autoaggression
20	Picardi et al., 2003	Cross-sectional	40	116 (other dermatological)	Alexi	Self-report	Higher alexithymia
21	Innamorati et al., 2016	Cross-sectional	100	97 (healthy)	Emo Alexi	Self-report	The effect of psoriasis on quality of life is mediated by difficulties in emotion regulation, anxiety, depression, and food craving Higher alexithymia
22	Talamonti et al., 2017	Cross-sectional	250	215 (healthy)	Alexi	Self-report	Higher alexithymia Association between alexitimia and female gender and involvment of sensitive body areas
23	Korkoliakou et al., 2014	Cross-sectional	100	100 (healthy)	Emo Alexi	Self-report	Higher alexithymia
24	Korkoliakou et al., 2017	Cross-sectional	108	Absent	Emo Alexi	Self-report	Psoriasis with alexithymia is related to higher somatization, interpersonal sensitivity, anxiety, and phobic anxiety than Psoriasis witout alexithymia
25	Consoli et al., 2006	Longitudinal	92	Absent	Alexi	Self-report	Patients with low emotional awareness are more reactive to stress and more responsive to treatment
26	Vari et al., 2013	Cross-sectional	33	33 (healthy)	ER	Self-report	Higher use of emotion supression
27	Ciuluvica et al., 2019	Cross-sectional	91	101 (healthy)	ER	Self-report	Higher use of emotion supression More impulse control difficulties, and non-acceptance of emotional responses
28	Ciuluvica et al., 2014	Cross-sectional	23	18 (dermatological) 27 (healthy)	ER	Self-report	Suppression is negatively related with quality of life, while reappraisal is positively related with patients well being
29	Almeida et al., 2017	Cross-sectional	228	Absent	ER	Self-report	Higher difficulties in ER negatively correlates with treatment satisfaction and positively correlates with: discomfort due to the disease; psychopathological symptoms; missed work/school days
30	Picardi et al., 2005	Cross-sectional	33	73 (dermatological)	Alexi ER	Self-report	More likely to have alexithymia. Lower perceived social support and higher insicure attachment
31	Larsen et al., 2017	Longitudinal	163	Absent	Alexi ER	Self-report	Lower self-management is associated with higher alexithymia
32	Mastrolonardo et al., 2006	Cross-sectional	25	50	ER	Behavioral	Higher increase of heart rate and diastolic blood pressure during stress induction
33	Mastrolonardo et al., 2007	Cross-sectional	25	50	ER	Behavioral	No change in cortisol levels and stress perception after stress induction
34	Panasiti et al., 2019	Cross-sectional	16	17	ER	Behavioral	Patients perform better and show reduced sympathetic system activity when the cognitive load associated to the emotional task is high
35	Jose and Menon, 2017	Cross-sectional	10	10 (acne) 10 (melanoma)	Stress	Self-report	More sensitive to stress
36	Simonić et al., 2013	Cross-sectional	45	191 (dermatologic)	Stress	Self-report	Psoriatic arthritis report less positive and more negative (stressful) life events during late childhood
37	O’Leary et al., 2004	Cross-sectional	141	Absent	Stress	Self-report	Perceived stress is associated with a poorer level of quality of life, higher levels of anxiety and depression

## Emotional Reactivity

Emotional reactivity is the constellation of behavioral and physiological changes triggered by the evaluation of a given situation in relation to one’s own active goals (Gross and Jazaieri, 2014). It can assume the form of a discrete emotion (i.e., an intense and short-lived response; Sander, 2013) or a feeling (i.e., the conscious experience of the emotion state; Tsuchiya and Adolphs, 2007), or it can be chronically altered in affective clinical disorders (i.e., fear in anxiety or sadness in depression). Pathological forms of emotional reactivity are typically characterized in terms of *emotion intensity* (e.g., emotional hyporeactivity), *emotion duration* (e.g., prolonged negative emotions), *emotion frequency* (e.g., frequent aggressive episodes), or *emotion type* (e.g., displaying inappropriate emotions) (Gross and Jazaieri, 2014). Psoriasis patients tend to experience a wide range of negative emotions that can be altered in several of these qualities (Sampogna et al., 2012). Below, we provide a detailed review of emotions (anger, disgust, and shame), feelings (stigmatization and social exclusion), and affective clinical disorders (anxiety and depression) that have been studied in relation to psoriasis.

### Anxiety

Psoriasis is characterized by anxiety (i.e., the feeling of apprehension, uncertainty, and fear) as showed by the high prevalence of anxiety disorders (13.1%; Lamb et al., 2017) diagnosed in these patients (Cepuch et al., 2014; Fleming et al., 2017). Self-reported anxiety seems to be higher in women with psoriasis with respect to men and is positively correlated with the severity of the disease (Pujol et al., 2013). Recently, higher level of anxiety and depression has been found in these patients, even in a sample of psoriasis patients with cognitive deficits (Innamorati et al., 2018).

### Stigmatization, Shame, and Disgust

Given its impact on patients’ physical appearance, psoriasis is often associated with a feeling of stigmatization, especially when it appears early in patients’ life (Schmid-Ott et al., 2007). Stigmatization is higher when the disease has an early onset and when the extent of bleeding and feeling of rejection are greater (Ginsburg and Link, 1989). It has been shown that high levels of stigmatization are caused by disease’s severity and, in turn, provoke a significant decrement of quality of life (Vardy et al., 2002). Moreover, stigmatization seems to (i) be the most powerful predictor of depressive symptoms in these patients (Hrehorów et al., 2012; Łakuta et al., 2017); (ii) be significantly related to psychological distress and degree of disability (Richards et al., 2001); and (iii) interfere with work and daily activities (Ginsburg and Link, 1993). Patients suffering from stigmatization tend not to have a partner, to have lower education, to have a higher level of social inhibition, to show a type D personality (van Beugen et al., 2017), to have higher stress and pruritus intensity, and to have lower quality of life (Hrehorów et al., 2012). In a recent study (Ponsi et al., 2019), we showed that in patients with psoriasis with respect to controls, higher sympathetic system activation during an experimental paradigm designed to induce the feeling of social exclusion (i.e., cyberball paradigm) was related to a higher need for social reconnection (i.e., the need to invest in new social interactions).

When chronic stigmatization is associated with an anxious ambivalent attachment style, dermatological patients’ view of themselves can be severely influenced, and they can manifest negative feelings of self-disgust (Jafferany and Patel, 2019). Psoriasis patients show higher sense of skin-related shame and disgust, which correlates with a less positive evaluation of being touched by their parents when they were kids (Lahousen et al., 2016). Interestingly, it has been shown that not only psoriasis patients but also their significant ones tend to avoid disgusted faces more than do controls (van Beugen et al., 2016). Shame—which is associated with the severity of psoriasis symptoms and also with depression and anxiety—seems to be higher in women than men, and it is more frequent in patients with a low level of education (Sampogna et al., 2012).

### Depression

It has been shown that the risk of developing depression in psoriasis patients (prevalence of 9.9% of Major Depressive Disorder; Lamb et al., 2017) seems to be mediated by the presence of other comorbidities, except in younger patients with severe psoriasis where the presence of the disease directly predicts the onset of depression (Jensen et al., 2016). Psychological distress, negative beliefs about one’s own appearance, and lower levels of emotional and social support are factors that predispose to the development of depression in psoriasis (Wojtyna et al., 2017). Also, compared with patients with other dermatological conditions such as acne or alopecia areata, psoriasis patients show higher scores of depression, and suicidal ideation (Pompili et al., 2016).

### Anger

It has been shown that in dermatologic conditions, aggression is associated with anxiety, and with a lower level of optimism and social support (Coneo et al., 2017). In psoriasis, anger (subclinical condition) frequency correlates with severity and length of the disease, and it is higher in patients with a low level of education (Sampogna et al., 2012). Psoriasis patients are characterized by a higher level of trait anger respect to controls; moreover, when they have low self-esteem, they show more anger toward people or objects and have enhanced difficulties in anger control (Aydin et al., 2017); conversely, they score very low in autoaggression (Matussek et al., 1985). Notably, however, one study reported that psoriasis patients exhibited fewer verbal aggression responses after anger-inducing procedures (Niemeier et al., 1999).

## Alexithymia

Alexithymia is a subclinical trait defined by difficulties in the following: (i) identifying, describing, and communicating one’s own feelings; (ii) differentiating them from emotionally unrelated bodily sensations; (iii) emotional awareness related to psychosomatic symptoms; and (iv) imagination, daydreaming, and introspection (Martin and Pihl, 1985; Taylor et al., 1991). Crucially, identifying emotions is believed to be related to the ability to regulate them (Chen et al., 2011).

Neuroscientific evidence links alexithymia to (i) aberrant emotion processing (i.e., decreased activation of limbic structures in response to negative emotional stimuli and angry vs. neutral faces; Kano et al., 2003; Van der Velde et al., 2013; (ii) reduced gray matter volume in emotional processing brain areas (Xu et al., 2018); and (iii) reduced connectivity within the default mode network (DMN), in brain areas involved in emotional awareness and increased connectivity of the DMN with areas involved in sensory input and emotion control (Liemburg et al., 2012).

The association between alexithymia and various medical disorders suggests that it may represent a risk factor for their development, probably because it enhances stress responses through autonomic dysregulation (i.e., the alexithymia–stress hypothesis; Martin and Pihl, 1985). In particular, alexithymic people seem not to cope effectively with stressors because of a stress response that is typically altered in its cognitive (i.e., lack of emotional awareness), behavioral (i.e., maladaptive coping and lack of emotional expression), and physiological (i.e., increased arousal) components (Martin and Pihl, 1985). This altered response to stress might prolong the exposure to stressors and, on the long run, exacerbate the somatovisceral response (Martin and Pihl, 1985).

Also, alexithymia presents hypo-reactive physiological responses rather than hyper-reactive ones (Van der Velde et al., 2013) and seems to be associated with poorer interoception and the tendency to misattribute bodily signals (Palser et al., 2018). Misinterpretation of bodily sensations associated with negative emotions might be another mechanism through which alexithymia worsens clinical conditions (Lumley et al., 1996; Tuzer et al., 2011).

Alexithymia is often associated with psoriasis (Picardi et al., 2003, 2005; Innamorati et al., 2016) (prevalence of 24.8%, Sampogna et al., 2017), especially in women and in cases in which the plaques extend to sensitive body areas (like the face, the hands, or the genitals) (Talamonti et al., 2017). These patients show a higher level of somatization, interpersonal sensitivity, anxiety, and phobic anxiety respect to non-alexithymic patients (Korkoliakou et al., 2014, 2017). Some researchers suggested that alexithymia might be a condition that patients acquired in order to avoid dealing with unwanted emotions (Panayiotou et al., 2015). Consistently with this point of view, emotional awareness, an emotional skill distinct but often correlated to alexithymia, consisting in the ability to integrate and differentiate emotions, predicts better response to treatment in psoriasis patients (Consoli et al., 2006). The reported studies measured alexithymia using the Toronto Alexithymia Scale (TAS−20; Bagby et al., 1994).

## Emotion Regulation

Emotion regulation is a multi-componential process that comprehends the implicit and explicit strategies through which we act on the emotional experience in order to enhance or reduce it (Gross and John, 2003). Maladaptive emotion regulation is a component of many psychopathological diseases such as depression (Ehring et al., 2010) and post-traumatic stress disorder (McLean and Foa, 2017).

Compared with controls, patients with psoriasis are characterized by higher use of emotional suppression (Vari et al., 2013; Ciuluvica et al., 2014, 2019), an emotion regulation strategy considered rather primitive that consists in inhibiting the expression of the ongoing emotional response once it has been generated (Gross and John, 2003). Interestingly, this is the same strategy used by recovered-depressed patients (Ehring et al., 2010). Conversely, higher use of reappraisal (Ciuluvica et al., 2014), an emotion regulation strategy that is more adaptive than suppression and consists in re-thinking the situation to alter its meaning and emotional impact (Gross and John, 2003), has shown to be positively related with patients’ well-being (Ciuluvica et al., 2014). In patients with psoriasis, higher difficulty in emotion regulation, as measured by the difficulty in emotion regulation strategies (DERS) scale, negatively correlates with treatment satisfaction and positively correlates with (i) the discomfort due to the disease; (ii) the number of reported psychopathological symptoms; and (iii) the frequency of missed work/school days (Almeida et al., 2017). Moreover, subtypes of psoriasis patients also show different patterns of emotion regulation: early-diagnosed patients have higher difficulties in behaving according to their goals when distressed (Almeida et al., 2017); obese patients with psoriasis show higher difficulties respect to obese patients without psoriasis (Innamorati et al., 2016). It has also been shown that the ability of impulse control (subclinical condition) when experiencing negative emotions is lower in this condition (Innamorati et al., 2016). Two subscales of the DERS, namely, emotional clarity and emotion acceptance, which are believed to measure concepts that are very close to alexithymia, also show higher scores among these patients (Innamorati et al., 2016). In agreement, in two recent studies, we showed that psoriasis patients scored higher than controls in the “Lack of Emotional Clarity” subscale of the DERS, indicating that patients have more difficulties than controls in correctly identifying their own emotions (Panasiti et al., 2019; Ponsi et al., 2019).

It has been hypothesized that low abilities in emotion regulation in psoriasis patients might increase the impact of poor social support on the severity of the disease (Picardi et al., 2005). Moreover, lower self-management, a psychological construct composed of medical management, role management, and emotional management, is associated with higher alexithymia in patients with moderate to severe psoriasis (Larsen et al., 2017).

It has to be noticed that most of the studies present in the scientific literature, at least to our knowledge, employed self-report measures or questionnaires. The lack of behavioral and physiological evidence regarding emotion regulation deficits in this population is crucial. Two studies reported some indirect measure by submitting patients to a standardized stressful procedure (mental arithmetic and the Stroop Color-Word Naming Test). They found higher heart rate and diastolic blood pressure in psoriasis patients (Mastrolonardo et al., 2006), which, however, was not accompanied by differences in stress perception or salivary cortisol levels (Mastrolonardo et al., 2007). Importantly, we recently showed that when presented with a working memory task with emotional distractors (i.e., the Emotional N-Back), psoriasis patients perform better and show reduced sympathetic system activity when the cognitive load associated with the task is high versus low and thus found it easier not to pay attention to the emotional distractors (Panasiti et al., 2019).

To sum up, the impact of emotion regulation abilities on the course of psoriasis seems crucial: patients’ well-being is negatively associated with suppression and is positively associated with reappraisal. Suppression and rumination are indeed more strongly linked to psychopathological outcomes than reappraisal and acceptance strategies (Kobylińska and Kusev, 2019). To our knowledge, there are no studies exploring the employment of acceptance strategies in psoriasis.

### Stress Managing

The experience of stress can impact each of the three aspects of emotional processing that we mentioned in this review (i.e., emotional reactivity, alexithymia, and emotion regulation): (i) exposure to stressors is correlated to higher experience of negative emotions (Feldman et al., 1999); (ii) higher basal cortisol level during stress anticipation is associated with higher alexithymia (de Timary et al., 2008); and (iii) acute stress impairs emotion regulation during fear conditioning (Raio et al., 2013).

Stress managing is pivotal in psoriasis patients because impaired emotional processing could affect not only the response to stressful events but also the quality of the general emotional response in psoriasis. Patients with psoriasis are more sensitive to stress with respect to other dermatological conditions such as acne or melanoma (Jose and Menon, 2017), and patients with psoriatic arthritis report less positive and more negative (stressful) life events during late childhood (Simonić et al., 2013). Stressful events are indeed very often reported by patients as the cause of the appearance or the exacerbation of the disease (Griffiths and Richards, 2001). Perceived stress in patients is significantly associated with a poorer level of quality of life and higher levels of depression and anxiety (O’Leary et al., 2004) and might be associated with dermatological worsening of the plaques (Basavaraj et al., 2011).

## Conclusion and Future Directions

From our review, it is apparent that emotional reactivity, alexithymia, and emotion regulation have a profound impact on the management of psoriasis symptoms. On the one hand, emotional reactivity in patients with psoriasis seems to be characterized by negative emotions such as anger (Matussek et al., 1985; Sampogna et al., 2012; Aydin et al., 2017), shame (Sampogna et al., 2012; Shah and Bewley, 2014; Lahousen et al., 2016), disgust (Lahousen et al., 2016), and feelings like social exclusion (Vardy et al., 2002; Schmid-Ott et al., 2007; Lahousen et al., 2016; van Beugen et al., 2017; Łakuta et al., 2017) and also by psychopathological disorders such as anxiety (Pujol et al., 2013; Cepuch et al., 2014; Fleming et al., 2017) and depression (Jensen et al., 2016). This emotional pattern seems to affect slightly more women (Sampogna et al., 2012; Pujol et al., 2013; Talamonti et al., 2017) than men and to be a risk factor for a wide range of negative outcomes spanning from lower quality of life (Vardy et al., 2002; O’Leary et al., 2004; Vari et al., 2013) to suicide (Pompili et al., 2016).

On the other hand, the ability to regulate emotions seems to be a protective factor that improves quality of life (Vari et al., 2013), treatment satisfaction, and the impact of negative emotions (Almeida et al., 2017). This is especially true when patients do not suffer from alexithymia. The effect of presence of alexithymia or low emotional awareness in these patients is not completely clear: on the one hand, it seems to help them in ignoring unwanted emotions (Panayiotou et al., 2015) and improve the treatment outcome (Consoli et al., 2006); on the other hand, it seems to worsen the impact of emotions on quality of life (Picardi et al., 2005; Almeida et al., 2017). From this literature review, it appears clear that treatments for psoriasis should also include techniques that address emotional reactivity, alexithymia, and emotion regulation because affective symptoms, together with dermatological ones, play a fundamental role in the resolution of this condition. One promising candidate would be the emotion regulation therapy (ERT), which is a manualized intervention that aims at (i) increasing emotional and motivational awareness; (ii) developing emotion regulation abilities; and (iii) generating new learning experiences (Renna et al., 2017).

Our review also highlights some limitations of the approaches that have been used so far for studying emotional processes in psoriasis. First of all, only few studies (Mastrolonardo et al., 2006, 2007; van Beugen et al., 2016; Panasiti et al., 2019; Ponsi et al., 2019) reported behavioral and physiological evidence. Although we acknowledge that self-report measures are important to understand the conscious evaluation that patients have of themselves, we also believe that implicit measures are crucial to understand what are the abilities that are truly compromised in these patients. Future studies should include these measurements and compare them with self-report measures in order to obtain a fine-grained picture of emotional processing in these patients. Second, many studies (15 of the 37 we reviewed) did not test a control group; this practice does not allow to disentangle whether what is observed is specific of this skin condition or is also true in the general population. Furthermore, very few studies tested a clinical control group with other dermatological conditions. Including such control groups would be very important to understand the altered psychological mechanisms behind psoriasis and to define efficient psychological treatments.

## Author Contributions

All authors listed have made a substantial, direct and intellectual contribution to the work, and approved it for publication.

## Conflict of Interest

The authors declare that the research was conducted in the absence of any commercial or financial relationships that could be construed as a potential conflict of interest.
